# Influence of Selenium: Lemire et al. Respond

**DOI:** 10.1289/ehp.1003242R

**Published:** 2011-04

**Authors:** Mélanie Lemire, Donna Mergler

**Affiliations:** Axe santé des populations et environnementale, Centre de recherche du Centre hospitalier universitaire de Québec, Québec, Québec, Canada, E-mail: Melanie.Lemire@crchuq.ulaval.ca; Centre de recherche interdisciplinaire sur la biologie, la santé, la société et l’environnement, Université du Québec à Montréal, Montréal, Québec, Canada

We agree with the comments made by Minoia et al. in their letter. In this study population, we did evaluate the sentinel signs and symptoms of selenosis (Lemire M, Philibert A, Fillion M, Passos CJS, Guimarães JRD, Barbosa F Jr, Mergler D, unpublished data), and we observed no association between any of the biomarkers of selenium (Se) status and signs and symptoms of selenosis (hair loss, broken nail walls, nail sloughing, skin lesions, garlic breath, gastrointestinal disorders, and motor and sensory deficits), despite high Se body burdens. Other results in this study population show a positive association between Se status and motor performance (Lemire M, Fillion M, Frenette B, Passos CJS, Guimarães JRD, Barbosa F Jr, Mergler D, unpublished data).

Guidelines on Se toxicity (International Programme on Chemical Safety 1986; U.S. Environmental Protection Agency 2002) are based mostly on reports of chronic selenosis in a Chinese population with excessive Se exposure, resulting from high Se in crops fertilized with coal ash highly rich in Se (Yang et al. 1983), and from combustion of Se-rich coal for domestic use (Liu et al. 2007). Drinking water likewise contained unusually high concentrations of inorganic Se (Yang et al. 1983). Thus, several factors, such as exposure to toxic vapors from coal combustion and/or inorganic Se from drinking water, may have contributed to toxic Se effects observed in China, primarily attributed to high organic Se in local crops. Recent studies reporting selenosis most often refer to excessive Se intake from nutritional supplements or inorganic Se through drinking water (Sutter et al. 2008; Vinceti et al. 2010).

Our study (Lemire et al. 2010) was conducted in 2006, before the first publication on an association between Se supplementation and the incidence of self-reported type 2 diabetes (Stranges et al. 2007). In the Amazonian population in our study, only 5 of the 448 participants reported diabetes. Further studies should address the possible associations between high Se status and hypertension and hypercholesterolemia (reviewed by Stranges et al. 2010).

Other evidence comes from Inuit, whose traditional diet of marine mammals is exceptionally rich in Se. The prevalence of diabetes in Nunavik, Québec, Canada, is low (3.5%). Ferland et al. (2009) observed no association between Se status and diabetes or plasma fasting glucose and insulin levels. Inverse association between blood Se and systolic blood pressure has also been reported (Valera et al. 2009). Hansen et al. (2004) pointed out that there are no recorded signs of selenosis in Greenland populations and that their high Se intake may be tolerated at higher levels.

In Northern and Amazonian populations exposed to mercury (Hg), high dietary Se may offset Hg-mediated oxidative stress and/or be required to maintain optimal selenoenzymes. There may be consequently less “excess” Se and little or no Se toxicity (Khan and Wang 2009).

Whether dietary Se is less toxic in Hg- exposed populations remains unanswered. However, even if there is increasing evidence that Se can offset some toxic effects of Hg, it may be inefficient against all Hg-mediated effects. Preventive actions should continue to focus on reducing Hg exposure rather than increasing Se status.
